# Non-invasive neurostimulation in OCD – implications for widescale application.

**DOI:** 10.1192/j.eurpsy.2023.108

**Published:** 2023-07-19

**Authors:** N. A. Fineberg

**Affiliations:** Life and medical science, University of Hertfordshire, Hatfield, United Kingdom

## Abstract

**Abstract:**

Brain-imaging findings implicate aberrant cortico-striatal neurocircuitry in the underlying pathology of OCD, so representing a potential treatment target. Ablative neurosurgery or deep brain (invasive) stimulation (DBS) of tracts or nodes within this circuitry is sometimes found to improve OCD, possibly by enhancing information-processing functions. Non-invasive neurostimulation, targeting superficial cortical nodes within cortico-striatal circuitry, is a safer and more acceptable alternative, with potential for scaling up and applying to a larger patient population, earlier in the course of illness.

Repetitive transcranial magnetic stimulation (rTMS) is the form of neuromodulation studied most in OCD. The orbitofrontal cortex (OFC), dorsolateral prefrontal cortex and supplementary motor area (SMA) have been identified as promising targets. The effect is larger for those not resistant to SSRI or failing to respond to only one SSRI trial. Thus, r-TMS may be best implemented earlier in the care pathway. rTMS is also relatively costly, involves specialist technical equipment and staff, and cannot be delivered in patients’ homes.

Transcranial direct current stimulation (tDCS) involves applying a low-amplitude (1-3mA) electric current to the brain via electrodes placed on the scalp. Compared with rTMS, tDCS tends to electrically modulate a more diffuse and superficial brain area, but it could represent a preferable option for patients with common mental disorders such as OCD, as it is cheaper, portable, simple and safe to use. In a recent randomised controlled feasibility study, the L-OFC represented the optimal target based on clinical changes. Further investigation of tDCS in OCD is warranted, to determine the optimal stimulation protocol, longer-term effectiveness and brain-based mechanisms of effect. If efficacy is substantiated, home-based approaches represent a rational next step.

**Disclosure of Interest:**

N. Fineberg Grant / Research support from: In the past three years I have received research funding from the NIHR, COST and Orchard; . I have additionally received financial support to attend meetings from the British Association for Psychopharmacology, European College for Neuropsychopharmacology, Royal College of Psychiatrists, International College for Neuropsychopharmacology, World Psychiatric Association, International Forum for Mood and Anxiety Disorders, American College for Neuropsychopharmacology. In the past I have received funding from various pharmaceutical companies for research into the role of SSRIs and other forms of medication as treatments for OCD., Consultant of: payment for expert advisory work on psychopharmacology for the Medicines and Healthcare Products Regulatory Agency, royalties from Oxford University Press and an honorarium from Elsevier for editorial work., Paid Instructor of: payment for lectures for the Global Mental Health Academy.

